# Integrating P4 Medicine in Teledentistry and M-Health in Oral, Dental, and Periodontal Care

**DOI:** 10.3390/jpm13010111

**Published:** 2023-01-04

**Authors:** Federica Di Spirito

**Affiliations:** Department of Medicine, Surgery and Dentistry, University of Salerno, 84084 Salerno, Italy; fdispirito@unisa.it

Given that dental practice is currently based on the “average” patient, providing therapeutic and rehabilitative interventions rather than preventive measures [1], with little consideration of individual characteristics and factors in disease developments and treatment responses [2], the profound incorporation of the P4 approach and technological advances in oral care may be of great benefit to both oral and interconnected general health.

P4 medicine, including precise (effective, efficient, and safe), personalized (targeted and individualized), preventive (early detection and intervention), and participatory (directly involving patients) approaches, is based on a deeper understanding of the etiology and course of oral disease and, in particular, the broader social, behavioral, and systemic determinants of oral health and its relationship to overall health [3].

Therefore, the increasing integration of P4 medicine principles into oral care should favor the transition from “reactive” disease-waiting dentistry with standardized protocols to “proactive” approaches that track patients’ parameters in a way that is as comprehensive, continuous, and prompt as possible [4].

In recent decades, strategies for P4 medicine have been developed, supported by digital technologies (including software and devices for data computation and storage) and medical data systems (especially “omics” data and those provided by patients and social networks), primarily to predict what will happen to a patient or a particular organ or site [4].

In oral, dental, and periodontal care, predicting the case-specific development of caries [5] or periodontitis [6] could support targeted primary prevention interventions that focus on individual characteristics and reduce the impact of individual modifiable risk factors [7]. From this perspective, the P4 dentistry approach could guide the provision of case-specific interventions and supportive care intervals to prevent disease onset and deterioration, with favorable relapses to oral and systemic health [8,9].

In addition, predicting the site-specific activity and progression of caries and periodontitis could support early-treatment decisions and more accurate, effective, and definitive approaches, with a positive impact on the thorough treatment of patients and their well-being [10].

Moreover, with the proliferation of technology and teledentistry [11], it is now possible to remotely collect and record a wide variety of patients’ clinical, imaging, and laboratory data records to define individualized diagnostic, prevention, and treatment pathways. Such data can also be easily shared across telehealth networks to enable the more comprehensive management of complex cases in multidisciplinary settings.

Teledentistry platforms and tools are increasingly used for consultations with urgent oral and dental needs and concerns, as well as for telediagnoses, especially for the elderly, the frail, and those living in remote areas [12,13,14,15].

Smartwatches, smartphones, and tablets are increasingly becoming an integrated part of our approach to health. M-health applications enable the progression of caries lesions and the progress of orthodontic treatment to be monitored, reinforce biofilm control and provide support and guidance for post-surgical courses [16].

Furthermore, teledentistry and M-health, in combination with widely used smart sensors on the one hand and artificial intelligence and machine learning on the other, may foster the development of next-generation systems [1,17] for automatic data collection and disease detection [1,17], further enhancing P4 strategies in oral, dental, and periodontal care. Integrating P4 medicine in teledentistry and M-health, as well as through next-generation systems [1,16], may potentially enable the early observation and detection of abnormalities [18,19,20], improve clinical decision-making, increase the effectiveness of clinical treatments by tailoring them to the individual patient [1,21,22,23] increase efficiency, and expand access to care, all while reducing distance and cost, in turn increasing prevention, especially in chronic disease management [18,19].
